# Sonolytic and Silent Polymerization of Methacrlyic Acid Butyl Ester Catalyzed by a New Onium Salt with *bis*-Active Sites in a Biphasic System — A Comparative Investigation

**DOI:** 10.3390/molecules18022419

**Published:** 2013-02-21

**Authors:** Perumberkandgai A. Vivekanand, Maw-Ling Wang, Yu-Ming Hsieh

**Affiliations:** 1 Department of Safety, Health and Environmental Engineering, Hungkuang University, Shalu District, Taichung City 43302, Taiwan; 2 Department of Health Nutrition and Biotechnology, Asia-Pacific Institute of Creativity, Toufen, Miaoli 35153, Taiwan

**Keywords:** ultrasonic irradiation, phase transfer catalysis (PTC), polymerization, kinetics, multi-site phase transfer catalyst, bi-phase system

## Abstract

Currently, ingenious new analytical and process experimental techniques which are environmentally benign techniques, *viz.*, ultrasound irradiation, have become immensely popular in promoting various reactions. In this work, a novel soluble multi-site phase transfer catalyst (PTC) *viz.*, 1,4-bis-(propylmethyleneammounium chloride)benzene (BPMACB) was synthesized and its catalytic efficiency was assessed by observing the kinetics of sonolytic polymerization of methacrylic acid butyl ester (MABE) using potassium persulphate (PPS) as an initiator. The ultrasound–multi-site phase transfer catalysis (US-MPTC)-assisted polymerization reaction was compared with the silent (non-ultrasonic) polymerization reaction. The effects of the catalyst and various reaction parameters on the catalytic performance were in detail investigated by following the kinetics of polymerization of MABE in an ethyl acetate-water biphasic system. From the detailed kinetic investigation we propose a plausible mechanism. Further the kinetic results demonstrate clearly that ultrasound-assisted phase-transfer catalysis significantly increased the reaction rate when compared to silent reactions. Notably, this environmentally benign and cost-effective process has great potential to be applied in various polymer industries.

## Abbreviations and Notations

US-MPTC: Ultrasonic assisted multi-site phase transfer catalysis
Silent-MPTC: Silent (Non-ultrasonic assisted) multi-site phase transfer catalysis
MABE: methacrlyic acid butyl ester
BPMACB: 1,4-bis-(propylmethyleneammounium chloride)benzene
PPS: potassium persulphate
MPTC: multi-site phase transfer catalyst
PTC: Phase transfer catalysis
µ: Ionic strength
H^+^: Acidic
R_p_: Rate of polymerization

## 1. Introduction

Currently about 40% of the synthetic rubber and 45% of the manufactured plastic materials are obtained by free radical polymerization processes. This class of polymerization has found myriad applications, including the manufacture of chemical surfactants [1] lubricants, thermoplastic block copolymer elastomers [2] (which may be used for a wide variety of applications including adhesives, footwear, and toys), polystyrene and cardiovascular stents [3].

In heterogeneous reaction systems, transformation of immiscible reactants into industrially desired compounds under mild reaction conditions has dual significance in view of both environmental protection and sustainable chemistry [4,5]. Phase transfer catalysis (PTC) is the one of the most promising methodologies in such biphasic reaction systems [6]. Among the various types of these catalysts, quaternary ammonium salts (such as Bu_4_NBr, Et_4_NBr, *etc.*) are the most frequently employed phase transfer catalysts [7,8,9]. The most significant applicationsof PTC is in the alkylation of benzyl cyanide and its derivatives, because many pharmaceuticals contain the phenylacetic framework [10,11,12].

Nowadays, PTC has become an important choice in organic synthesis [13] and is widely applied in the manufacturing processes of specialty chemicals, such as pharmaceuticals, dyes, perfumes, additives for lubricants, pesticides, and monomers for polymer synthesis. The applications of PTC have many salient features with respect to economics, operational simplicity and environmental consciousness in modern organic synthesis due to which PTC chemistry has been widely recognized as one of the most potent methodologies supporting green chemistry [14].

Although vast numbers of single-site phase transfer catalysts are available, however their selection mainly depends on the scale of economy. Since the single site catalysts do not meet the requirement of economy and efficiency; researchers are becoming interested in synthesizing multi-site phase-transfer catalysts [15,16,17,18,19,20,21,22,23]. These types of poly-active sites based salts meet the requirement of cost-effectiveness and increased efficiency owing to their multiple active sites per molecule. They are relatively easy to prepare and more importantly, the total weight of the multi-site PTC required in a particular reaction is less compared to that of a related single-site catalyst. Classical phase transfer catalysts [Q^+^X^−^] contain s single active site and hence they can transport/ferry just one anion from the reactant molecules [M^+^Y^−^] in the aqueous phase to organic phase during a catalytic cycle in the form of catalytic intermediate [Q^+^Y^−^]. However, when multi-site phase transfer catalysts are employed, multiple numbers of intermediates can be ferried from the aqueous phase into the organic phase in a catalytic cycle.

One of the branches of chemistry that has seen vast development in last couple of decades due to PTC is polymer chemistry [24,25,26,27]. Potential application of polymers in industrial, technological and biomedical fields are well documented [28,29]. Recently, such polymers have also considered as candidate materials in advanced fields such as information technology, electric and electronic science and biomedicine. Phase transfer-catalyzed free radical polymerization of monomers has several advantages compared to azobisisobutyronitrile assisted polymerization [30].

Free radical polymerization of olefinic monomers under phase transfer catalysis conditions was reported for the first time by Rasmussen and Smith [31]. They explored polymerization of butyl acrylate using various crown ethers and quaternary ammonium salts as phase transfer catalysts in ethyl acetate/water bi-phase systems using peroxydisulfate as the initiator. In the presence of phase transfer catalysts, peroxydisulfate can be phase transferred into a variety of solvents, including hydrocarbon solvents, with surprising facility, though it was known that the phase transfer of divalent anions like S_2_O_8_^2−^ was “notoriously slow” and as such could be utilized for the rapid polymerization of methacrylic and acrylic monomers, even at ambient temperatures. They postulated that the formation of quaternary peroxydisulfate (Q_2_^+^S_2_O_8_^2−^) which was soluble in the organic medium was responsible for the rapid monomer polymerization. The superior rate of decomposition of S_2_O_8_^2−^ to sulfate ion radicals in the presence of phase transfer catalysts caused the facile polymerization.

The poly(methyl methacrylates) prepared by phase transfer catalyzed polymerization [31] were found to have high molecular weight and more uniform molecular weight distribution than the polymers prepared by conventional organic initiators such as AIBN under similar reaction conditions. A thorough kinetic investigation of PTC-catalyzed free radical polymerization of MMA using K_2_S_2_O_8_ as water-soluble initiator and triethylbenzylammonium chloride as PTC was reported by Balakrishnan and Jayachandramani [32]. Recently, some attempts have been made to explore applications of MPTC in polymerization reactions [33].

Currently the invention of selective, efficient and eco-friendly methods for applications in complex organic synthetic manipulations constitutes a major chemical research effort. In this regard, several non-conventional methods are emerging that involve reactions in aqueous media [32] or those that are accelerated by exposure to microwave [33,34,35] or ultrasound [36,37,38] irradiation. These methods are now recognized as viable environmentally benign alternatives [33,34,35,36,37,38]. Although, sonication methods have been initially applied to homogeneous reactions in a variety of solvents, this approach has now evolved into a useful technique in heterogeneous reactions. A vast majority of sonochemical applications in the synthesis deal with reactions involving metals [39,40,41] organic phase insoluble reagents, or their aqueous solutions [39,42,43]. Compared with the traditional methods, a large number of reactions can be carried out in higher yields, shorter reaction time and milder reaction conditions under ultrasonic irradiation [44,45,46,47,48,49,50]. Sonochemical studies on the effect of ultrasonic on the polymer synthesis have been reported [51,52]. However, little investigation on the ultrasonic-multi-site phase transfer catalyzed (US-MPTC) polymerization has been found.

**Scheme 1:**
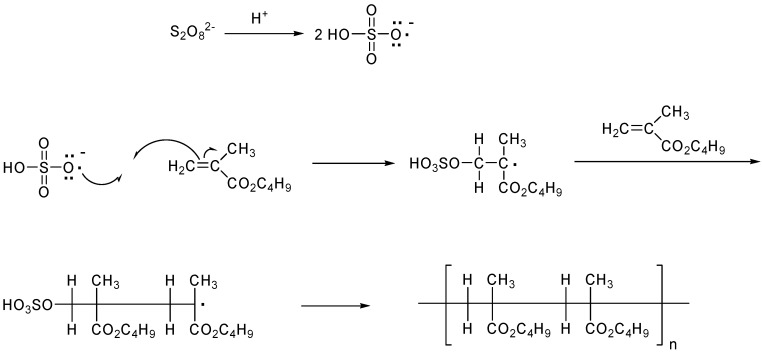
Mechanism of phase transfer-catalyzed free radical polymerization.

In continuation of our interest in the application of ingenious techniques in conjunction with phase transfer catalysis [53,54], we report herein for the first time the application of simultaneous ultrasound and phase transfer catalysis technologies in the polymerization reaction of methacrlyic acid butyl ester (MABE) catalyzed by newly synthesized bis-onium salt *viz.*, 1,4-bis-(propylmethyleneammounium chloride)benzene (BPMACB) using potassium persulphate (PPS) as water soluble initiator (Scheme 1). The polymerization was carried out in a specially designed ultrasonic reactor designed by Ko Hsieh Instruments Co. Ltd., Taipei, Taiwan. To our best knowledge, this is the first reported polymerization of methacrlyic acid butyl ester carried out in the presence of ultrasonic irradiation. The influence of different parameters such as concentration of MABE, concentration of PPS, concentration of BPMACB, concentration of ionic strength, concentration of [H^+^], kinds of organic solvents, different ultrasonic frequency, volume fraction of aqueous phase and operating temperatures have been studied in detail. Further, one of the salient features of current investigation is the comparison of polymerization process in silent (non-ultrasonic) and ultrasonic conditions.

## 2. Results and Discussion

### 2.1. Proposed Free Radical Polymerization Mechanism for MABE

Under phase transfer catalysis conditions, methacrlyic acid butyl ester (MABE) may be polymerized *via* free-radical polymerization, a type of chain growth reaction. Initially, the catalytic intermediate (QS_2_O_8_) is generated from the reaction of catalyst and initiator:

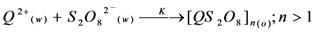
(1)

Three steps are involved in the free-radical polymerization [55] *viz.*, chain initiation, in propagation, and chain termination. In this work, we emphasized the rate of chain propagation step, R_p_ which is well known to the polymerization processes as shown in Equation (2).

The equation of the rate of propagation is represented by:

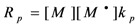
(2)

**Figure 1:**
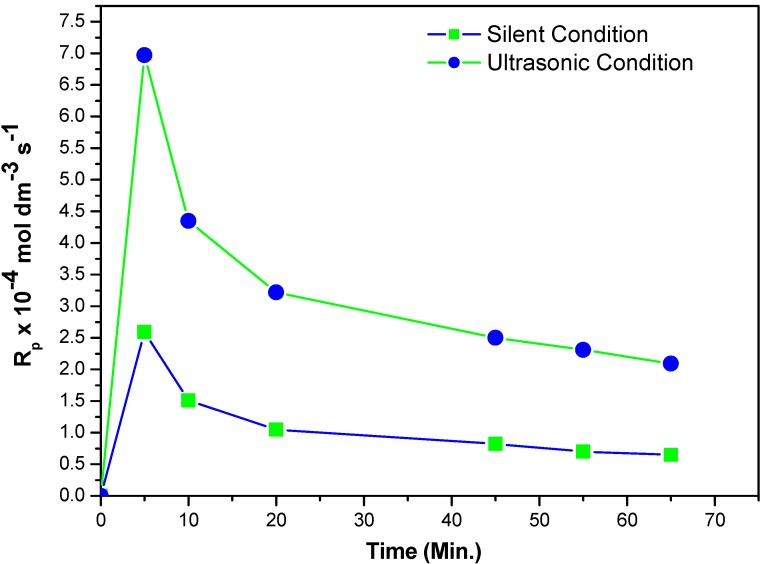
A plot of R_p_ *vs.* time, indicating steady-state rate of polymerization of MABE: 2.00 × 10^−2^ mol·dm^−3^ of PPS, 1.2 × 10^−2^ mol·dm^−3^ of BPMACB, 3.0 mol·dm^−3^ of MABE, 0.3 mol·dm^−3^ of [H^+^], 0.1 mol·dm^−3^ of [µ], 60 °C of temperature (50 kHz, 300 W for ultrasonic assisted reaction).

For the purposes of catalytic evaluation of the new catalyst, its catalytic utility was examined in the conventional polymerization reaction of MABE using PPS as an initiator under ultrasonic irradiation and silent conditions in ethyl acetate-water biphase at 60 °C. Identification of the steady state of approximation was achieved by carrying out the polymerization at various time intervals *viz.*, 5, 10, 20, 45, 55, 65 min. From the plot of Rp *vs.* time (Figure 1), it is obvious that the steady state rate of polymerization was attained after 45 min. Hence, we followed the kinetics of polymerization by fixing the reaction time as 45 min to carry out the variations in other reaction parameters *viz.*, concentration of MABE, concentration of PPS, concentration of BPMACB, concentration of ionic strength, concentration of [H^+^], kinds of organic solvents, different ultrasonic frequency, volume fraction of aqueous phase and operating temperatures.

### 2.2. Effect of the Ultrasound Frequency on Rate of Polymerization

The choice of ultrasonic frequency is very important in terms of both physical and chemical effects. Hence it has been the subject of much research and discussion [56] for many years and it is the underlying driving force for sonochemistry. In recent times, we found that ultrasound irradiation enhances rate of solid-liquid phase-transfer catalysis (SLPTC) bi-phase system [53]. Ultrasound-assisted PTC leads to more optimum reaction conditions than either of the two techniques alone [57]. In such cases, the phase-transfer catalyst initiates the reaction by the transfer of species across the interface and ultrasound merely facilitates this transfer, possibly by increasing the interfacial area across which this transfer occurs. Nowadays, this technique is recognized as a viable, environmentally benign technique to conduct chemical reactions.

**Figure 2:**
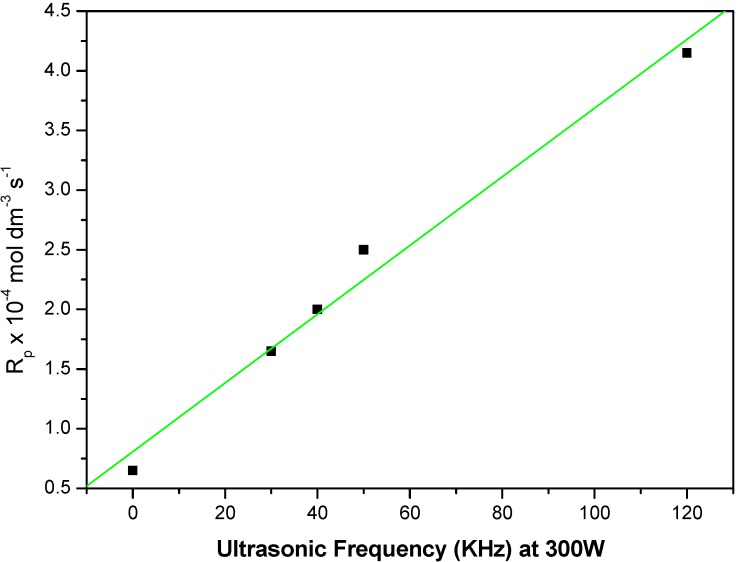
Influence of ultrasonic frequency variation on the rate of polymerization of MABE catalyzed by BPMACB: 2.00 × 10^−2^ mol·dm^−3^ of PPS, 1.2 × 10^−2^ mol·dm^−3^ of BPMACB, 3.0 mol·dm^−3^ of MABE, 0.3 mol·dm^−3^ of [H^+^], 0.1 mol.dm^−3^ of [µ], 60 °C of temperature.

In the present work, we investigated the effect of ultrasound frequency on the rate of polymerization of MABE. This was achieved by varying ultrasonic frequencies from 28–120 kHz and keeping ultrasonic power at 300 Watts under otherwise similar conditions using BPMACB (MPTC) as the catalyst. Also we followed the reaction under silent condition. The kinetic profile of the reaction is obtained by plotting log R_p_ *vs.* US frequency (Figure 2) taking the data at reaction time 45 min.

In our experiments a model L-400 ultrasonic bath was used. It consists of two layers stainless steel bodiesy. The internal dimensions of the ultrasonic cleaner tank are 250 mm × 250 mm × 380 mm with a liquid holding capacity of 23 liters. The external tank size is 420 mm × 520 mm × 600 mm. Further details are presented in the Experimental section. Under silent conditions without ultrasonic irradiation the rate of polymerization (R_p_) is only 0.82 × 10^−4^ mol·dm^−3^·s^−1^, but in the presence of ultrasonic the R_p_ values are found to be: 1.59 × 10^−4^ mol·dm^−3^·s^−1^, 2.01 × 10^−4^ mol·dm^−3^·s^−1^, 2.50 × 10^−4^ mol·dm^−3^·s^−1^ and 4.12 × 10^−4^ mol·dm^−3^·s^−1^ for 28, 40, 50 and 120 kHz, respectively. From these observed results, it can be inferred that ultrasonic assisted phase-transfer catalysis significantly increased the rate of the reaction. This could be ascribed to two facts, which are explained as follows:

(i) Ultrasonic irradiation causes cavitation and heating [58]. Once microscopic cavitation bubbles collapses at the surface of the substrate, they generate powerful shock waves that cause effective mixing of the layers. This feature of cavitation phenomena is an important factor is significant in heterogeneous reaction systems compared to homogeneous systems because the effect of cavitation is several hundred times greater in heterogeneous systems than in homogeneous systems. When the ultrasonic waves propagate in a liquid solution, the alternate compression and depression are produced to form cavities [59].
(ii) Further, it was evident that the application of ultrasound has resulted in an intensification of the overall interfacial area where the mass transfer of the reactive species takes place.

As a result, the rate of ultrasonic reaction has increased compared to the silent reaction [54,60,61].

### 2.3. Effect of Organic Solvents

**Figure 3:**
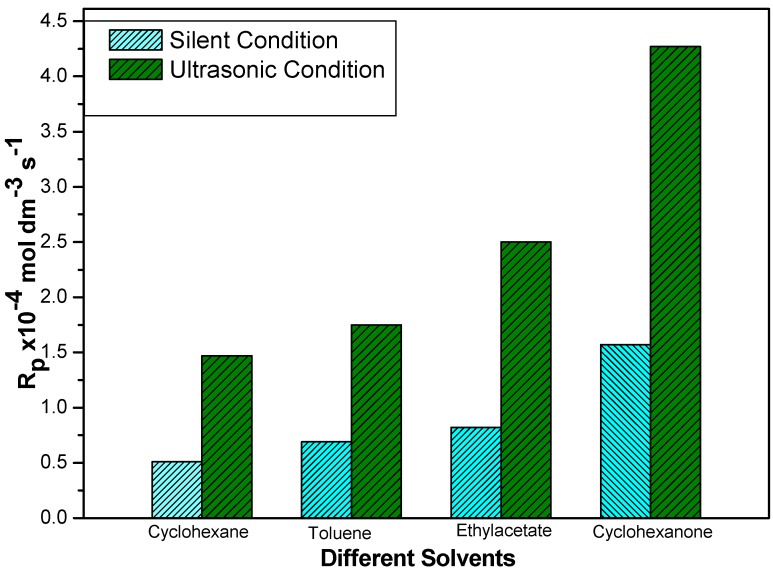
Rate of the polymerization of MABE under the US-MPTC and Silent-MPTC conditions in relation to the different solvents: 2.00 × 10^−2^ mol·dm^−3^ of PPS, 1.2 × 10^−2^ mol·dm^−3^ of BPMACB, 3.0 mol·dm^−3^ of MABE, 0.3 mol·dm^−3^ of [H^+^], 0.1 mol·dm^−3^ of [µ], 60 °C of temperature (50 kHz, 300 W for ultrasonic assisted reaction).

In this work, the influence of various organic solvents on the rate of polymerization of MABE was followed under otherwise standard reaction conditions. Four organic solvents employed in this study are cyclohexanone, toluene, cyclohexene and ethyl acetate. From Equation (2), the rate of polymerization values are obtained. The order of the reactivity for these five organic solvents is: cyclohexanone (C_6_H_12_O_1_) > ethyl acetate (C_2_H_5_COOEt) > toluene (C_7_H_9_) > cyclohexene (C_6_H_10_). The effect of the organic solvents on the rate of polymerization (R_p_) is shown in Figure 3. The increase in R_p_ values is attributed to the increase in the dielectric constant of the organic solvent. We attribute this dependence of dielectric constant values to the following factors:

(i) Firstly, transfer of the ionic species in the current investigation, catalytic intermediate [QS_2_O_8_]_n_, from the aqueous phase to the organic phase is greatly enhanced as the dielectric constant value of organic solvent increases.
(ii) Secondly, as organic solvent’s dielectric constant value results in favourable separation of ions, which results in an increase in the rate of propagation.

Therefore, we believe that there will be an increase in the rate of polymerization with an increase in dielectric constant of the organic medium [62,63,64].

### 2.4. Effect of Initiator (PPS) on Rp under Ultrasonic Condition

**Figure 4:**
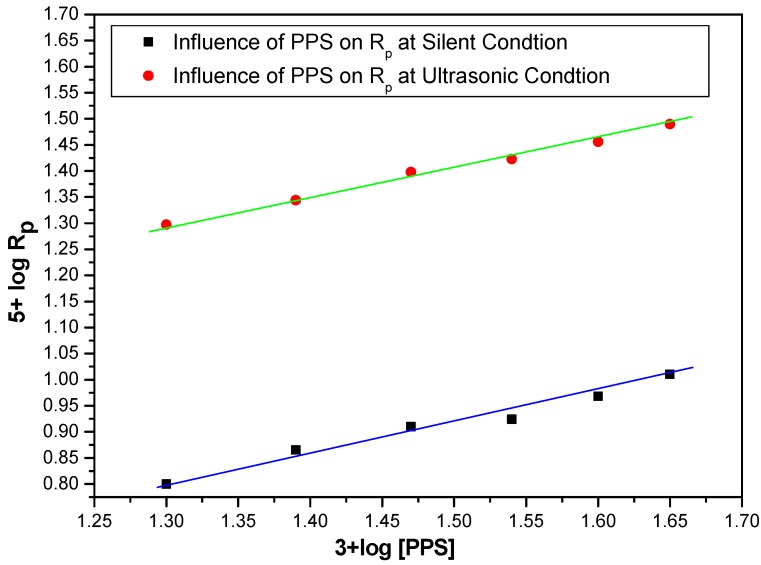
Bi-logarithmic plot illustrating influence of PPS concentration on the R_p_ for the polymerization of MABEunder US-MPTC and Silent-MPTC conditions: 1.2 × 10^−2^ mol·dm^−3^ of BPMACB, 3.0 mol·dm^−3^ of MABE, 0.3 mol·dm^−3^ of [H^+^], 0.1 mol·dm^−3^ of [µ], 60 °C of temperature (50 kHz, 300 W for ultrasonic assisted reaction).

**Figure 5:**
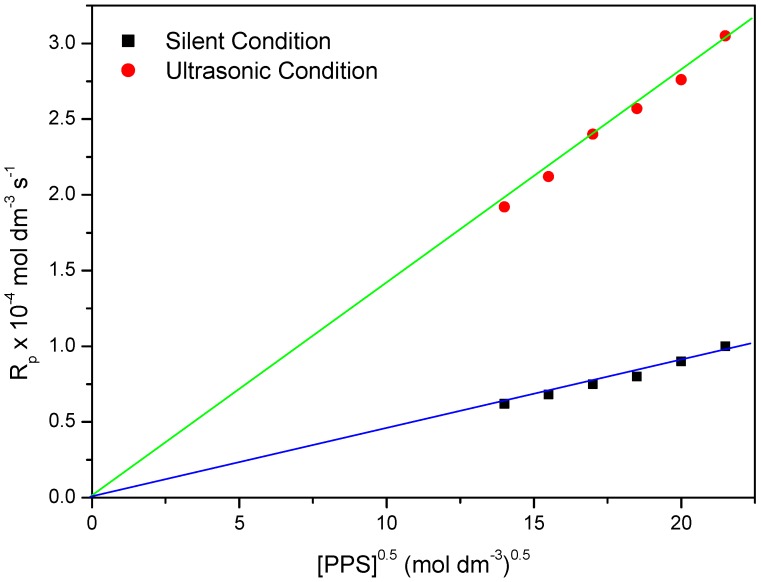
Plot of R_p_ *vs.* [PPS] under US-MPTC and Silent-MPTC conditions: 1.2 × 10^−2^ mol·dm^−3^ of BPMACB, 3.0 mol·dm^−3^ of MABE, 0.3 mol·dm^−3^ of [H^+^], 0.1 mol·dm^−3^ of [µ], 60 °C of temperature (50 kHz, 300 W for ultrasonic assisted reaction).

At a fixed monomer concentration MABE: 3.0 mol·dm^−3^, BPMACB: 1.2 × 10^−2^ mol·dm^−3^, [H^+^]: 0.3 mol·dm^−3^ and ionic strength: 0.1 mol·dm^−3^, the effect of PPS concentration on R_p_ was studied by varying the concentration of PPS in the range of 0.020–0.045 mol·dm^−3^ under ultrasonic conditions. The plot of (3 + log R_p_) *vs.* (5 + log [PPS]) was found to be linear, with a slope equal to 0.5, indicating the half order dependence on [PPS] (Figure 4). A plot of R_p_ *vs.* [PPS]^0.5^ was also found to be linear, passing through the origin (Figure 5), and supporting the half order dependence on [PPS].

In the free radical polymerization process, the order with respect to initiator is observed to be 0.5 wherever termination is bimolecular. An initiator rate exponent of 0.5 in the present study shows that the gel effect is not observed in this case. In the polymerization of styrene using PPS-tetrabutylammonium bromide system under silent condition, the order with respect to initiator 0.5 was reported [65].

### 2.5. Effect of Amount of BPMACB (Catalyst)

**Figure 6:**
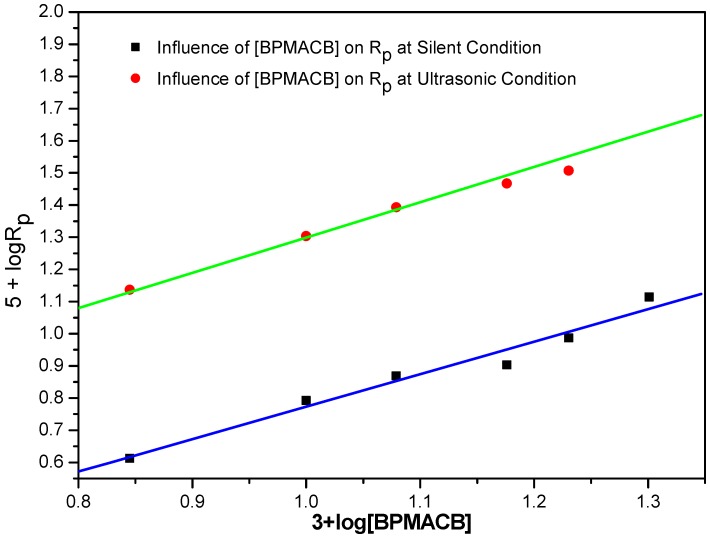
Bi-logarithmic plot illustrating the influence of BPMACB concentration on the R_p_ for the polymerization of MABE under US-MPTC and Silent-MPTC conditions: 2.00 × 10^−2^ mol·dm^−3^ of PPS, 3.0 mol·dm^−3^ of MABE, 0.3 mol·dm^−3^ of [H^+^], 0.1 mol·dm^−3^ of [µ], 60 °C of temperature (50 kHz, 300 W for ultrasonic assisted reaction).

**Figure 7:**
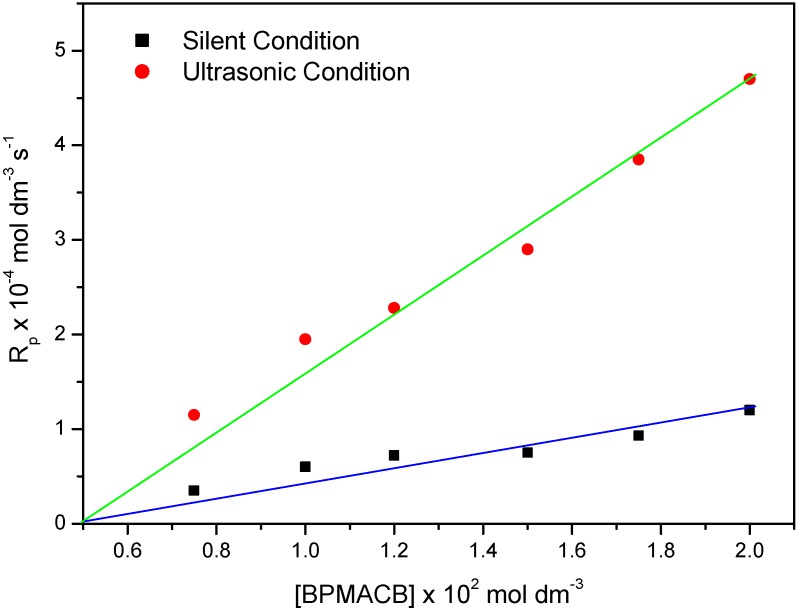
Plot of R_p_ *vs.* [BPMACB] under US-MPTC and Silent-MPTC conditions: 2.00 × 10^−2^ mol·dm^−3^ of PPS, 3.0 mol·dm^−3^ of MABE, 0.3 mol·dm^−3^ of [H^+^], 0.1 mol·dm^−3^ of [µ], 60 °C of temperature (50 kHz, 300 W for ultrasonic assisted reaction).

The effect of the amount of BPMACB on the conversion is given in Figure 6. It is clear that the polymerization rate is enhanced by the addition of the new *bis*-onium catalyst. From the slope of the linear plot obtained by plotting (3 + log Rp) *vs.* (5 + log [BPMACB]), the order with respect to QX_2_ was found to be 1.0. The observed order of 1.0 was confirmed from the straight line passing through the origin in a plot of Rp *vs.* [BPMACB] (Figure 7). The increase in the rate of polymerization values due to increase in the amount of catalyst used, reveals three factors that are involved in the current variation study:

(i) Availability of higher number of active-sites due to increase in the amount of catalysts [9,17].
(ii) Increase in opportunity of collision between initiator and catalyst molecules.
(iii) Concentration of catalytic intermediate increases in the reaction medium intermediate by increasing catalyst concentration [17,54].

### 2.6. Rate of Polymerization (R_p_) and Methacrlyic Acid Butyl Ester (MABE Monomer) Concentration

**Figure 8:**
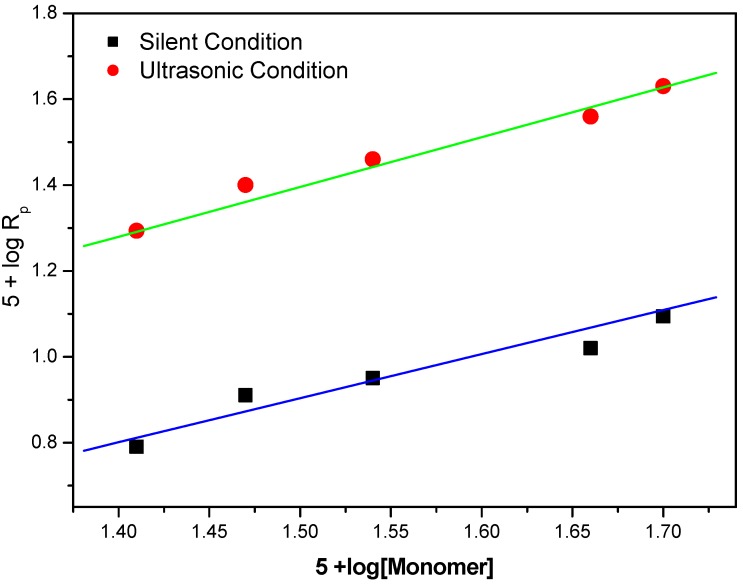
Bi-logarithmic plot illustrating influence of PPS concentration on the R_p_ for the polymerization of MABE under US-MPTC and Silent-MPTC conditions: 2.00 × 10^−2^ mol·dm^−3^ of PPS, 1.2 × 10^−2^ mol·dm^−3^ of BPMACB, 0.3 mol·dm^−3^ of [H^+^], 0.1 mol·dm^−3^ of [µ], 60 °C of temperature (50 kHz, 300 W for ultrasonic assisted reaction).

The effect of monomer concentration on the rate of polymerization was studied by varying the monomer concentration and at fixed concentration of potassium persulphate (PPS): 0.02 mol·dm^−3^ and BPMACB: 0.012 mol·dm^−3^ (Figure 8).

**Figure 9:**
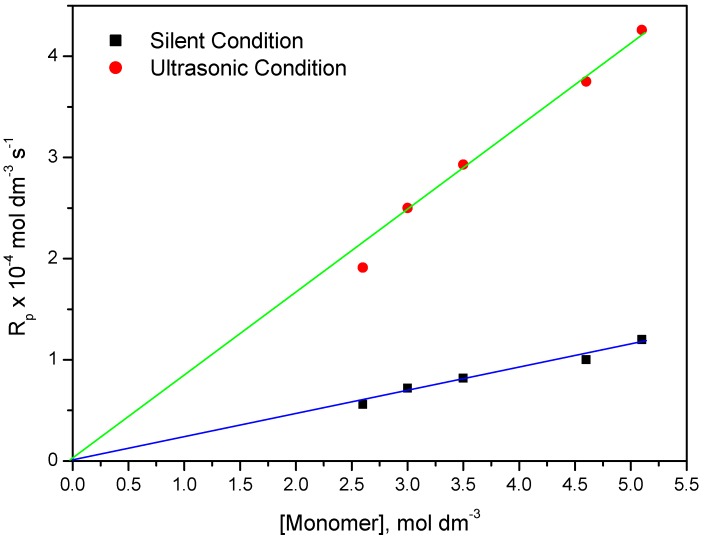
Influence of [MABE] on the rate of polymerization catalyzed by BPMACB: 2.00 × 10^−2^ mol·dm^−3^ of PPS, 1.2 × 10^−2^ mol·dm^−3^ of BPMACB, 0.3 mol·dm^−3^ of [H^+^], 0.1 mol·dm^−3^ of [µ], 60 °C of temperature (50 kHz, 300 W for ultrasonic assisted reaction).

A plot of (5+log(Rp)) *vs.* (5+log [MABE]) was found to be linear (Figure 8) with slope of unity clearly indicating the dependence of Rp on [MABE] [33]. *i.e.*, R_p_ was found to increase with increase of [MABE]. The reaction order of 1.0 with respect to MABE is further confirmed from the straight line passing through the origin in the plot of R_p_ *vs.* [MABE] (Figure 9).

### 2.7. Dependence of Rp on Ionic Strength and H^+^

Under standard reaction condition, the effect of ionic strength (µ) and [H^+^] were observed in the range of 0.3–0.1 mol·dm^−3^ [K_2_SO_4_] and 0.4–0.1 mol·dm^−3^, respectively. These variations do not exert any significant change in the R_p_ values in the present investigation. Similar insignificant dependence of Rp on concentration of ionic strength and H^+^ are reported in the literature [32,55].

### 2.8. Effect of Temperature on Rp

**Figure 10:**
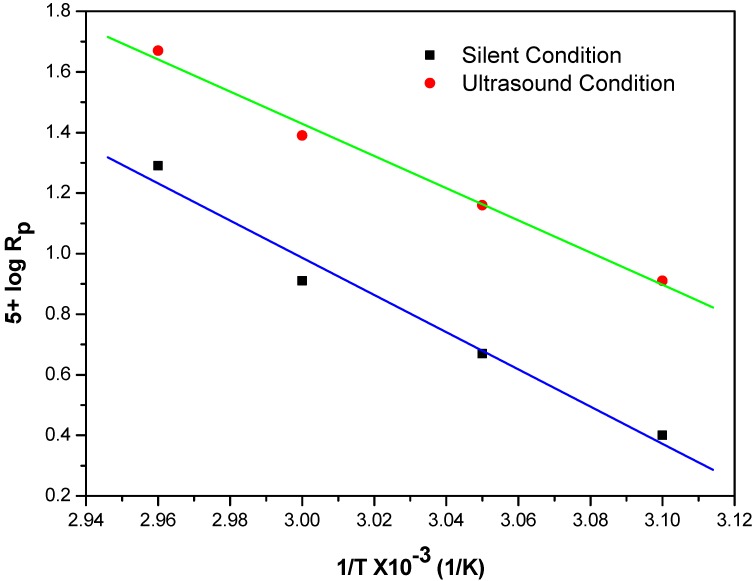
Arrhenius plot for the polymerization of MABE under the US-MPTC and Silent-MPTC conditions: 2.00 × 10^−2^ mol·dm^−3^ of PPS, 1.2 × 10^−2^ mol·dm^−3^ of BPMACB, 3.0 mol·dm^−3^ of MABE, 0.3 mol·dm^−3^ of [H^+^], 0.1 mol·dm^−3^ of [µ] (50 kHz, 300 W for ultrasonic assisted reaction).

To ascertain the influence of temperature on the R_p_, the temperature was varied from 50–65 °C under unstirred ultrasonic and silent condition. As expected, the R_p_ value increases with the corresponding increase in temperature values. The activation energy was calculated from the Arrhenius plot of log Rp *vs.* 1/T (Figure 10); E_a_ = 14.40 kcal/mol (Silent-MPTC) and 12.24 kcal/mol (US-MPTC). Previously, we reported similar lower activation energy for ultrasonic assisted reactions compared to silent reactions [66,67].

The result is consistent with results obtained previously in studies of different PTC reaction systems [68,69]. It is evident that the rate of polymerization increases with an increase in the temperature along with ultrasonic effect [54]. We attribute this phenomenon to following factors:

(i) Number of cavitation bubbles, *i.e.*, reaction loci, increases as temperature increases;
(ii) Increase in collision of the reactants molecules at higher temperature. Consequently, the reaction rate is increased at higher temperature.

### 2.9. Effect of Ratio of Volume of Water to Volume Organic Solvent (V_w_/V_o_)

**Figure 11:**
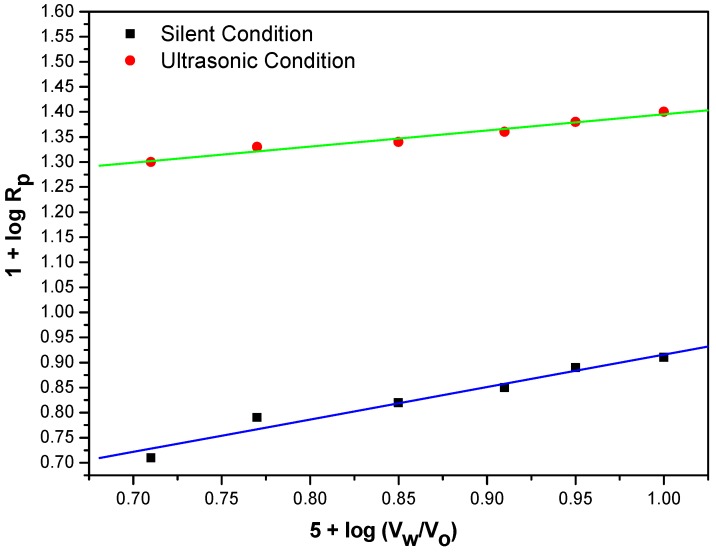
Bi-logarithmic plot illustrating influence of volume fraction of aqueous phase on the R_p_ for the polymerization of MABE under US-MPTC and Silent-MPTC conditions: 2.00 × 10^−2^ mol·dm^−3^ of PPS, 1.2 × 10^−2^ mol·dm^−3^ of BPMACB, 3.0 mol·dm^−3^ of MABE, 0.3 mol·dm^−3^ of [H^+^], 0.1 mol·dm^−3^ of [µ], 60 °C of temperature (50 kHz, 300 W for ultrasonic assisted reaction).

To investigate the influence of ratio of volume of water to volume organic solvent (V_w_/V_o_) on the kinetics of polymerization of MABE, their ratio was varied from 0.52 to 1.0 under sonocatalyzed and silent reaction standard reaction conditions. The R_p_ values were found to increase only slightly (Figure 11). In the free radical polymerization of glycidyl methacrylate catalyzed by phase transfer catalyst similar small increase was reported [33].

## 3. Experimental

### 3.1. Materials and Instruments

Potassium persulphate, tripropylamine, hydroquinone, ethyl acetate, cyclohexanone, toluene and other reagents are all reagent-grade chemicals from ACROS (Geel, Belgium), Aldrich (Steinheim, Germany), Echo (Miaoli, Taiwan), Fluka (Buchs, Switzerland), Lancaster (Cambridge, UK), Merck (Darmstadt, Germany), SHOWA (Tokyo, Japan), TCI (Tokyo, Japan), TEDIA (Fairfield, OH, USA). These were used as received without any further purification. Methacrylic acid butyl ester (Lancaster) was first washed with aqueous sodium hydroxide (5%) to remove the inhibitor, washed with water to remove the alkali, dried over anhydrous calcium chloride and distilled in the presence of dry oxygen-free nitrogen under reduced pressure. The middle fraction of the second distillate was collected and stored in dark brown bottle at 5 °C in the refrigerator. The NMR spectra were recorded on a Mercury 400 NMR spectrometer. The FT-IR spectra were recorded on a Perkin–Elmer Spectrum 1000 spectrophotometer.

Ultrasonic energy is transmitted to the process vessel through the liquid medium, usually water in the tank. For safety purpose, the sonochemical reactor consisted of two layers stainless steel body. The sonochemical reactor configuration used in the present work is basically an ultrasonic bath (model L-600) equipped with flat transducer, procured from Ko Hsieh Instruments Co. Ltd., (Taipei, Taiwan) (http://www.kohsieh.com.tw/). The external tank size is 420 mm × 520 mm × 600 mm. The internal dimension of the ultrasonic cleaner tank is 250 mm × 250 mm × 380 mm with liquid holding capacity of 23 liters. The reactor has an operating frequency of 25 kHz and 50 kHz with an output of 300 W. Both ultrasounds are separately produced through a flat transducer mounted at the bottom of the tank. In this ultrasonic instrument there is a provision for a drain as well as an outlet at the top, which allows continuous operation of work. Further, in order to carry out high and low temperature reactions, an additional heater (Thermo Haake, BRAMHS GmbH, Germany) with temperature controller facility has been provided. The process vessel is immersed in the water present in the tank.

### 3.2. Synthesis of 1,4-bis-(propylmethyleneammounium chloride)benzene, BPMACB

1,4-Bis-(chloromethyl)benzene (5.0 g, 0.02 mol) was dissolved in ethanol (50 mL) and transferred into a 150 mL capacity three-necked flask. The solution was deaerated by flushing a stream of nitrogen followed by addition of tripropylamine (4.50 g, 0.03 mol) to the solution. The reaction mixture was gently refluxed for 24 h under nitrogen atmosphere and then cooled to room temperature. Organic solvent was stripped in a vacuum evaporator. A crude product is washed with hexane (3 × 10 mL) and recrystallized from ethanol to obtain the desired solid MPTC (BPMACB). The catalyst was identified by FT-IR, ^1^H-NMR and ^13^C-NMR.

### 3.3. Sonolytic Kinetics of the Bi-Phase Reaction System

Polymerization kinetic measurements were carried out in a biphasic reaction mixture consisting of aqueous (10 mL) and organic phase (10 mL each), *i.e.*, total volume of the reaction mixture is 20 mL, in the temperature range 40–60 °C (±0.1 °C) under dearation condition (N_2_). Known quantities of organic phase, monomer (MABE) and solvent (ethyl acetate), were taken in a reactor and flushed with purified nitrogen gas to ensure inert atmosphere. To the organic phase, known quantities of catalyst (BPMACB) was added. In order to maintain ionic strength and [H^+^] strength of the reaction mixture, known quantities of potassium sulfate sulfuric acid were added and the biphasic reaction mixture was flushed with purified nitrogen gas for about 30 min to ensure an inert atmosphere. The reaction vessel was suspended into the ultrasonic microwave bath. A calculated amount of deaerated potassium persulphate (PPS) solution thermostated at the experimental temperature was added to the reaction mixture. The mixture was sonicated (50 kHz, 300 W for ultrasonic assisted reaction) and simultaneously a stop watch was started. The reaction tube was then carefully sealed by rubber gaskets to ensure an inert atmosphere. On the addition of PPS to the reaction mixture, polymerization was initiated and precipitate formed continuously during polymerization. Samples were collected at regular intervals of time and the polymerization was arrested by pouring the reaction mixture into ice-cold methanol containing traces of hydroquinone. The polymer formed was filtered out quantitatively through a sintered crucible (IG-4), washed several times with double distilled water, methanol and dried at 50–60 °C in a vacuum oven to constant weight. The rate of polymerization, R_p_ was calculated gravimetrically [32,52] by substituting the weight of the polymer obtained and other parameters in each experiment in Equation (3)

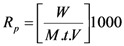
(3)
where V= Total volume of the reaction mixture, M = Molecular weight of the monomer used, t = Reaction time in seconds and W = Weight of the polymer. Polymer analyses were carried out by FT-IR and NMR.

## 4. Conclusions

The synergetic effect of a novel bis-onium salt and ultrasonic irradiation in the polymerization of methacrlyic acid butyl ester (MABE) is presented. The study reveals the rate polymerization (Rp) under ultrasonic irradiation condition is more efficient than under silent conditions. The factors affecting the reaction such as different ultrasonic frequencies, concentration of MABE, concentration of PPS, concentration of BPMACB, concentration of ionic strength, concentration of [H^+^], kinds of organic solvents, volume fraction of aqueous phase and different temperatures were investigated. The rate of polymerization increases with increasing concentration of catalyst, concentration of monomer, concentration of initiator, ultrasonic frequencies and temperature. Nevertheless, the ionic strength and hydrogen ion concentration of the medium do not show any substantial effect on the rate. The orders with respect to catalyst, initiator and monomer were found to be 1.0, 0.5 and 1.0, respectively. From the results obtained, a suitable kinetic scheme and mechanism has been proposed. Amalgamation of ultrasound and MPTC resulted in better efficacy as compared to the individual operations.
